# Side Effects of Pesticides and Metabolites in Groundwater: Impact on Denitrification

**DOI:** 10.3389/fmicb.2021.662727

**Published:** 2021-05-13

**Authors:** Caroline Michel, Nicole Baran, Laurent André, Mickael Charron, Catherine Joulian

**Affiliations:** ^1^BRGM, DEPA (Direction de l’Eau, de l’Environnement, des Procédés et Analyses), Orléans, France; ^2^Université d’Orléans, CNRS, BRGM, UMR 7327 Institut des Sciences de la Terre d’Orléans, Orléans, France

**Keywords:** denitrification, groundwater, chloroacetanilide, conazole, pesticides, metabolites, non-target effects

## Abstract

The impact of two pesticides (S-metolachlor and propiconazole) and their respective main metabolites (ESA-metolachlor and 1,2,4-triazole) on bacterial denitrification in groundwater was studied. For this, the denitrification activity and the bacterial diversity of a microbial community sampled from a nitrate-contaminated groundwater were monitored during 20 days in lab experiments in the presence or absence of pesticides or metabolites at 2 or 10 μg/L. The kinetics of nitrate reduction along with nitrite and N_2_O production all suggested that S-metolachlor had no or only little impact, whereas its metabolite ESA-metolachlor inhibited denitrification by 65% at 10 μg/L. Propiconazole and 1,2,4-triazole also inhibited denitrification at both concentrations, but to a lesser extent (29–38%) than ESA-metolachlor. When inhibition occurred, pesticides affected the reduction of nitrate into nitrite step. However, no significant differences were detected on the abundance of nitrate reductase *narG* and *napA* genes, suggesting an impact of pesticides/metabolites at the protein level rather than on denitrifying bacteria abundance. 16S rRNA gene Illumina sequencing indicated no major modification of bacterial diversity in the presence or absence of pesticides/metabolites, except for ESA-metolachlor and propiconazole at 10 μg/L that tended to increase or decrease Shannon and InvSimpson indices, respectively. General growth parameters suggested no impact of pesticides, except for propiconazole at 10 μg/L that partially inhibited acetate uptake and induced a decrease in microbial biomass. In conclusion, pesticides and metabolites can have side effects at environmental concentrations on microbial denitrification in groundwater and may thus affect ecosystem services based on microbial activities.

## Introduction

One consequence of the increasing use of pesticides is the presence of both parent molecules and transformation products (metabolites) in various environmental matrices and notably groundwater, with metabolites being even referred to as emerging groundwater contaminants (Jurado et al., 2012; Lapworth et al., 2012; Baran and Gourcy, 2013; Lopez et al., 2015). One drawback is that pesticides used in agriculture to target insects, fungi, or plants are now known to have side effects on non-target surface and subsurface living (micro)organisms (Iker et al., 2010; Staley et al., 2015). The environmental consequence of such side effects of pesticides and metabolites on microbial ecosystems is that they can threaten the ecosystem services based on microbial activities in soil (e.g., litter degradation, plant growth, nutrient cycling, and degradation of pollutants) and groundwater (e.g., production of drinking water, nutrient cycling, degradation of contaminant) (Tuinstra and van Wensem, 2014; Griebler and Avramov, 2015).

Most studies on the side effects of pesticides have been conducted in soils, and the main impacts were noticed on microbial abundance, presence or absence of microbial species, increase or decrease in gene expression (mainly linked to the N cycle), and increase or decrease of functional diversity [activities measured using the Biolog EcoPlates (C cycle) or soil activities linked to P, N, S, and C cycles] (Hussain et al., 2009; Lo, 2010; Kalia and Gosal, 2011; Yang et al., 2011; Jacobsen and Hjelmsø, 2014). Moreover, the presence of pesticides was shown to usually lead to the selection of microorganisms having the ability to degrade them (Bælum et al., 2008; Lancaster et al., 2010). The modes of actions of pesticides are multiple, which could explain the diversity of side effects observed on microbial communities and activities (Saez et al., 2003, 2006). In addition, the effects of pesticides on soil microbial ecosystems mainly depend on the type of pesticides and their concentration, as well as time after application. They also depend on the microbial community structure and on the diversity of the microbial processes that are taking place in the studied soil. Physical and chemical factors such as the type of soil, pesticide concentration, presence of organic matter, and adsorption and desorption processes also influence the impact of pesticides on microbial communities (Hussain et al., 2009). It was also demonstrated that some metabolites obtained after the biological and physicochemical transformations of pesticides can be more persistent and/or more toxic than the parent molecules (Bollag and Kurek, 1980). Taking all together, these factors increase the difficulty to evaluate the risks associated with pesticides use and to predict the net effects of pesticides on soil ecosystem health (Yang et al., 2011; Staley et al., 2015). In addition, contradictory conclusions on the impacts of pesticides can be found among studies due to the difficulty to compare results from different works done with great differences in experimental setups, pesticide concentrations, and methods (Jacobsen and Hjelmsø, 2014). The bioavailability/sorption/biodegradation of pesticides can also vary a lot for the same pesticides according to the studied system (Jacobsen and Hjelmsø, 2014).

The very few studies conducted in groundwater have also underlined the potential side effects of pesticides on groundwater microbial communities. Results first suggested that the presence of pesticides could increase microbial biodiversity. For instance, Imfeld et al. (2018) obtained higher Shannon and Simpson diversity indices (from T-RFLP fingerprints) for groundwater communities in batch experiments amended with pesticides (metolachlor) compared with experiments without the addition of pesticides. de Lipthay et al. (2004) also demonstrated that the diversity of colony morphotypes (culturable) in sediments from a subsurface aquifer exposed to herbicides (as mixture) was higher compared with the non-exposed samples. These authors also observed an increase of the relative abundance of bacterial heterotrophs in aquifer sediment exposed to pesticides, but no specific bacterial species were found for herbicide-exposed samples. Janniche et al. (2012) also noticed high diversity indices from microbial community-level physiological profiles (CLPP) using EcoPlates^TM^ in herbicide (isoproturon, atrazine, and acetochlor)-exposed groundwater. Studies, however, underlined the difficulties to determine *in situ* the impact of pesticides on groundwater biodiversity due to natural spatial and temporal variations of groundwater bacterial communities (de Lipthay et al., 2004; Imfeld et al., 2018). Moreover, they suggested that biodiversity modifications induced by pesticides were not associated to changes in general catabolic properties. The role of the contamination history of pesticides on its impact on groundwater microbial communities has also been underlined. As an example, Iker et al. (2010) showed that in an aquifer impacted with atrazine over a long time, a new contamination with this pesticide leads to the decrease of the relative abundance of *amoA* gene and nitrite-oxidizing bacteria, even if ammonia is the primary degradation product of atrazine. On the opposite, in an aquifer never impacted with atrazine before, atrazine spiking led to an increase in the relative abundance of nitrite-oxidizing bacteria. In another study, Mauffret et al. (2017) showed that a triazine concentration of 1 μg/L was enough to induce modification of the bacterial community structure in non-contaminated groundwater batch experiments, but a 10 times higher (10 μg/L) triazine concentration was needed to obtain the same impact in historically contaminated groundwater.

The possibility to extrapolate all the knowledge obtained for soil communities to groundwater ecosystems is still difficult to establish. The first reason is that the concentrations of pesticides in groundwater are low (in hundreds of nanograms to micrograms per liter order) compared with those found in soil, with metabolite contents usually higher than parent molecules (Amalric et al., 2013; Lopez et al., 2015). The second reason is that aquifers are physically, chemically, and biologically different from soil. Groundwater is indeed characterized by a quite constant temperature (around 12–14°C in temperate climates), no sunlight, and a low easily available nutrient content (low organic carbon and oxygen availability) (Griebler and Lueders, 2009; Gregory et al., 2014; Taubert et al., 2019; Retter et al., 2021). Aquifers can be connected to the surface but this connection greatly varies from an aquifer to another, and transfer rates can be so slow that some aquifers can be considered as isolated environments (Hubalek et al., 2016). All of this represents the most important differences with soils and can greatly influence microbial diversity and activities. In particular, lithoautotrophs that fix CO_2_ and oxidize inorganic electron donors as energy sources are an important part of groundwater microbial communities. Previous studies also support the idea that groundwater microbial diversity is different from that found in the overlying surface soil (Griebler and Lueders, 2009; Taubert et al., 2019) even if soil microorganisms can be transported into groundwater (Dibbern et al., 2014; Lazar et al., 2017). Moreover, groundwater is usually characterized by lower biodiversity and biomass than soil, and the presence of several novel microbial phyla has also been demonstrated (Griebler and Lueders, 2009; Anantharaman et al., 2016). Finally, the composition of microbial communities varies across aquifers, which is mainly due to species sorting imposed by local environmental conditions (mineralogy, water chemistry, etc.) as well as other factors such as dispersal limitation and drift across areas, type of aquifer and its connection to the surface, anthropogenic activities, etc. (Gregory et al., 2014; Fillinger et al., 2019; Sonthiphand et al., 2019). When considering all of this, and due to the low nutrient content of aquifers, it can be expected that the addition of exogenous organic compounds such as pesticides and/or their metabolites even at low concentration has impacts on the microbial community in terms of biodiversity and/or activity. This is particularly true as pesticides and metabolites are quite persistent (due to low or no biodegradation) and tend to persist in groundwater. Indeed, even if the potential biodegradation of pesticides in aquifers was already demonstrated (Janniche et al., 2012) with the isolation of pesticide-degrading bacteria, the biodegradation rates of pesticides in aquifers are significantly lower than those observed in topsoil (Hoyle and Arthur, 2000; Albrechtsen et al., 2001; Grenni et al., 2009; Barra Caracciolo et al., 2010).

Among the most used pesticides in Europe that are found in groundwater, chloroacetanilide pesticides, used for the control of annual weeds, mainly on corn, sugar beet, and sunflower, are frequently detected together with their transformation products, notably ethane sulfonic, and oxanilic acids (Kalkhoff et al., 2012; Amalric et al., 2013; Baran and Gourcy, 2013; Sidoli et al., 2016). Among the chloroacetanilide family, S-metolachlor, an herbicide used worldwide, is one of the top five pesticides detected in France [with a maximal concentration of 20.9 μg/L in Amalric et al. (2013) and 0.95 μg/L in Lopez et al. (2015)] and in the EU (Loos et al., 2010). Its metabolite ESA-metolachlor is also widely detected in French groundwater [at a maximum of 4.8 μg/L in Amalric et al. (2013) and with an average concentration of 0.21 μg/L in Baran and Gourcy (2013)]. Triazole fungicides, widely used on fruits, vegetables, and cereals, are also frequently detected in groundwater. During the French national campaign of 2012, propiconazole was one of the most quantified fungicides (Lopez et al., 2015). One of the main metabolites of all triazole fungicides is 1,2,4-triazole, also used in Europe as a nitrification and urease inhibitor. Few data exist on its surveillance in France: its presence was reported in surface waters (Scheurer et al., 2016), and no quantification was done in groundwater during the French national campaigns, which might be due to a high limit of quantification (0.1 μg/L). Recent publications of compiled groundwater monitoring in the United States demonstrate once again the occurrence of these molecules in groundwater and the significant levels that can be observed there. As examples, Bexfield et al. (2021), on a panel of 1,204 sites, measured maximum concentrations of 0.0237, 1.419, and 2.509 μg/L for propiconazole, metolachlor, and ESA-metolachlor, respectively, and Fisher et al. (2021) (54 sites) obtained concentrations of 0.0254, 0.075, and 4.04 μg/L for propiconazole, metolachlor, and ESA-metolachlor, respectively. Both studies also provided new data on 1,2,4-triazole with a maximum concentration of 0.296 μg/L for Bexfield et al. (2021) (with a detection frequency of 1.4%) and 0.436 μg/L for Fisher et al. (2021).

There is thus a need to study the impact of pesticides and metabolites on the microbial communities and the linked ecosystem services in groundwater. As mentioned above, pesticides/metabolites can affect the N cycle and thus microbial denitrification activity involved in ecosystem services such as the production of drinking water. The objective of this study was thus to evaluate in controlled conditions the impact of pesticides or metabolites on the potential denitrification activity and biodiversity of a groundwater microbial community. One herbicide (S-metolachlor) and one fungicide (propiconazole) belonging to two different chemical classes (respectively, chloroacetanilide and conazole) and their major metabolite ESA-metolachlor and 1,2,4-triazole, respectively, were selected. These two active substances were chosen due to their wide use in France and Europe and their occurrence in groundwater, as mentioned above. These four compounds were tested at two concentrations, 2 and 10 μg/L, similar to those measured in groundwater, in a batch experimental approach to investigate their impacts on groundwater microbial denitrification activity and biodiversity. Groundwater from an agricultural site historically submitted to nitrate use was chosen for this study. The denitrification activity was determined by following the evolution of nitrate, nitrite, and nitrous oxide concentrations, whereas variations of bacterial biodiversity between the start and the end of the experiment were determined thanks to genomic characterizations.

## Materials and Methods

### Chemicals

Molecules were provided as pure chemicals by Dr. Ehrenstorfer (S-metolachlor), Sigma Aldrich (ESA-metolachlor), and HPC Standards (propiconazole and 1,2,4-triazole). Individual concentrated solutions (500 mg/L) were prepared in methanol and then diluted in sterile water to obtain a 10-mg/L solution used to spike groundwater samples. Two final concentrations of pesticides and metabolites were used in this study: 2 and 10 μg/L.

### Field Site and Groundwater Sampling and Chemical Analyses

Sampling was done on a catchment located in the NW of France (Plourhan, French Brittany) (Petelet-Giraud et al., 2021; Surdyk et al., 2021). Several piezometers have been drilled in 2006 in the sector. The geological setting corresponds to an amphibolite (metamorphic rock, basement rock).

In the frame of a monitoring campaign in October 2017, a groundwater sample (8 L) was collected from piezometer Pz3 after discarding three purge volumes and stabilization of parameters measured *in situ* (temperature, pH, redox potential, electrical conductivity, and dissolved oxygen). This piezometer has a depth of 19 m and a water table level fluctuating between 2 and 4 m below soil level (bsl) in average and is equipped with a screen between 7 and 19 m bsl. For the analyses of anions and major cations, a sample was collected in 100 ml PE bottles after filtration through a 0.45-μm filter. The sample for major cation analyses was acidified to pH 2 with ultrapure nitric acid. All samples were cooled for transportation to the laboratory and stored at 4°C until chemical analysis. Pesticides and major ions were then analyzed at the laboratory (Table 1). Chemical analyses were performed by using ICP-AES for Ca^2+^, Na^+^, K^+^, Si^2+^, and Mg^2+^ (with 5% uncertainty); ion chromatography for Cl^–^, SO_4_^2–^, and NO_3_^–^ (with 10% uncertainty); colorimetric method based on NF ISO 15923-1 for NH_4_^+^, PO_4_^3–^, and NO_2_^–^; and potentiometric methods according to NF EN ISO 9963-1 (1996) for HCO_3_^–^ and CO_3_^2–^ (with 5% uncertainty). Dissolved organic carbon (DOC) was quantified according to NF EN 1484 (1997) procedures. For pesticides, a 55-pesticides monitoring (occurrence and concentration), including mother molecules and metabolites from pesticides degradation, was performed by liquid chromatography-mass spectrometry (LC-MS/MS) following an online solid-phase extraction (Amalric et al., 2013). The chemical analysis done on Pz3 groundwater validated previous results and showed that this piezometer is impacted by nitrate (78 mg/L) but not by pesticides or metabolites (Table 1).

**TABLE 1:** Water chemistry and physical properties of groundwater collected from well Pz3 in October 2017.

Water level (from topsoil) (m)	4
Temperature (°C)	12
pH (*in situ* measurement)	6.1
Conductivity (mS/cm)	455
Redox potential (Eh, mV)	223
Dissolved oxygen (mg/L)	1.8
Ca (calcium) (mg/L)	29
Cl (chloride) (mg/L)	50
HCO_3_ (bicarbonates) (mg/L)	61
CO_3_ (carbonates) (mg/L)	<LQ
K (potassium) (mg/L)	1.4
Mg (magnesium) (mg/L)	9.2
Ammonium (as NH_4_) (mg/L)	<LQ
Nitrite (as NO_2_) (mg/L)	<LQ
Nitrate (as NO_3_) (mg/L)	79
Na (natrium) (mg/L)	43
P (phosphate as PO_4_) (mg/L)	0.1
S (sulfur as SO_4_) (mg/L)	18
Si (silica as SiO_2_) (mg/L)	34
Dissolved organic carbon (DOC) (mg/L)	< LQ
Pesticides and metabolites (mg/L)	<LQ

*LQ, limit of quantification.*

### Groundwater Incubation (Batch Experiments)

To study the impact of pesticides and metabolites at two concentrations on denitrification in groundwater, pesticide/metabolite-spiked batch experiments were undergone in triplicate. Flasks (300 ml) were filled in with 150 ml groundwater (to obtain a gas/liquid ratio of 50/50) in anoxic conditions (nitrogen atmosphere). Taking into account nitrate concentration in the piezometer (Table 1), flasks were also supplemented with nitrate to reach a final NO_3_^–^ concentration of 100 mg/L. Pesticides and metabolites were added at a final concentration of 2 or 10 μg/L with the exception of positive control flasks (denitrification in the absence of pesticides/metabolites) and abiotic test flasks (addition of sodium azide) (Cabrol et al., 2017). Acetylene was added in the gas phase (10% acetylene/90% nitrogen) to inhibit the last step of the denitrification pathway, i.e., nitrous oxide (N_2_O) reduction into nitrogen gas, leading to N_2_O accumulation instead of N_2_ production (Milenkovski et al., 2010; Crouzet et al., 2016). Acetate (C_2_H_3_NaO_2_) was added as a carbon source in each flask at a final concentration of 100 mg/L. This initial acetate concentration was defined in order to cover the carbon needs of heterotrophic bacteria (catabolism and anabolism) during the denitrification process and to avoid any substrate limitation in relation with initial nitrate concentration (André et al., 2011). Flasks were incubated during 20 days in the dark at 25°C under stirring (100 rpm) to favor water contact with acetylene. At regular times (every 2–3 days), gas sampling (5 ml, using a Vacuette tube system) for N*_2_*O quantification and water sampling (8 ml) for nitrate, nitrite, and acetate quantification were done. To circumvent the depression linked to gas and water sampling, an equal volume of nitrogen (90%)/acetylene (10%) gas was added after each sampling. Nitrate and acetate were quantified by ionic chromatography (Dionex IC3000-SP-EG-DC system equipped with an AS50 autosampler and a conductimetric detector) according to the NF EN ISO 10304-1 (2009) method. Nitrite was analyzed by colorimetry according to the NF ISO 15923-1 (2014) method. N_2_O was analyzed by gas chromatography using a Varian CP-3800 GC equipped with a gas injection valve and an electron capture detector. Finally, after 20 days of incubation, 20 ml of water was sampled, filtered (0.22 μm), and stored at −20°C for molecular analyses.

### Molecular Analyses

Microbial DNA was extracted from frozen filters using the FastDNA^TM^ Spin Kit for Soil (MP Biomedicals, United States) according to the manufacturer’s recommendations with a FastPrep^®^-24 instrument at a speed of 5 ms^–1^ for 30 s and quantified using the Quantifluor dsDNA sample kit and the Quantus fluorimeter, according to the manufacturer’s instructions (Promega, United States). The abundance of the bacterial universal marker (16S rRNA gene) and of nitrate-reducing bacterial markers *narG* and *napA* genes was assessed by duplicated real-time quantitative PCR (qPCR). The reaction mixture contained 1× SSO Advanced Supermix (Bio-Rad), 0.4 μM of each primer, 100 ng of T4gp32 (MP Biomedicals), 2 μl of 0.03–0.5 ng/μl of template DNA, and qs 20 μl of nuclease-free water. For 16S rRNA gene, primers 341F (5′-CCTACGGGAGGCAGCAG-3′) and 515R (5′-ATTACCGCGGCTGCTGGCA-3′) (López-Gutiérrez et al., 2004; Crouzet et al., 2016) and the following thermocycling conditions were used: 95°C for 3 min, 35 cycles of 95°C for 30 s, 60°C for 30 s, 72°C for 30 s, and a data acquisition step at 80°C for 30 s at each cycle. For *narG* and *napA* genes, the respective primer sets narG-F (5′-TCGCCSATYCCGGCSATGTC-3′) and narG-R (5′-GAGTTGTACCAGTCRGCSGAYTCSG-3′) and V17m (5′-TGGACVATGGGYTTYAAYC-3′) and napA4r (5′-ACYTCRCGHGCVGTRCCRCA-3′) described in Bru et al. (2007) and the following thermocycling conditions were used: 3 min at 95°C; 6 cycles of 30 s at 95°C, 30 s at 63°C (*narG*) or 61°C (*napA*) with a touchdown of −1°C by cycle, 30 s at 72°C; 34 (*narG*) or 40 (*napA*) cycles of 30 s at 95°C, 30 s at 58°C (*narG*) or 56°C (*napA*), 30 s at 72°C, 30 s at 80°C. Standard curves were obtained from serial 10-fold dilutions of linearized plasmids containing known copy numbers of the target gene. No-template controls were run for each qPCR assay. qPCR was run in a CFX Connect Real-Time PCR Detection and data were analyzed with the CFX Manager 3.1 software (Bio-Rad).

Diversity of the bacterial community was determined by 16S rRNA gene Illumina sequencing. Amplicon libraries and sequences were generated by the MetaHealth CIRAD platform (Montpellier, France) using a modified version of the Illumina 16S “Metagenomic” Sequencing Library Preparation Protocol. Briefly, the 16S rRNA V3–V4 gene region was targeted for PCR amplification, in a nested PCR strategy using primers 341F (5′-CCTACGGGNGGCWGCAG-3′) and 785R (5′-GACTACHVGGGTATCTAATCC-3′) modified with Illumina-specific overhang sequences for barcoding. The first PCRs were run in duplicate using the Phusion Flash High-Fidelity PCR Master Mix (Thermo Fisher), and 10-fold diluted PCR products were then subjected to the second PCR to implement dual barcodes. DNA library was obtained after two successive purifications (Wizard^®^ DNA Clean-Up System kit, Promega) of the second PCR products. Library quantitation was performed by running the library on a D5000 ScreenTape bioanalyzer (Agilent). Sequencing was performed on an Illumina MiSeq platform with MCS v2.6.2.1. For Fastq generation, base calling and associated quality scores were done with Illumina RTA 1.18.54, and demultiplexing was done with MiSeq Reporter 2.6.2.3. Fastq quality control was evaluated with FastQC v0.11.7 (Andrews, 2010) and summarized with multiqc v1.5 (Ewels et al., 2016).

Fastq sequences were processed using the FROGS bioinformatics pipeline (Escudié et al., 2017) implemented into the GenoToul Galaxy platform (Afgan et al., 2018). In brief, after denoising and primer and adapter removal, paired reads were merged with VSEARCH and clustered into OTU with SWARM and an aggregation distance of 3. After chimera removal and filtering for OTU abundance (threshold of 0.00005%), taxonomic affiliation was performed using BLASTn and the 132 Silva database. Filtration on taxonomic affiliation was done at minimum identity of 98.2% and minimum coverage of 99%. Random resampling of the sequences obtained from the 24 samples was applied to have an equal number of 11,200 good-quality sequences per sample. The FROGS implemented Phyloseq R package was used for OTU structure visualization, rarefaction curve, and diversity index calculations. Shannon’s index (*H’*) (heterogeneity of microbial community: *H’* is minimal if all the individuals of a community belong to the same species, or if in a community, all the present species are represented by one individual except for one species which is represented by the remaining individuals of the community) and Simpson’s index (1/*D* in this paper: probability that two individuals picked at random do not belong to the same species; diversity is low if 1/*D* is low and vice versa) were calculated.

### Statistics

For statistical analyses, boxplots were calculated from triplicate flasks with data obtained during the 0–7-day period and corresponding to the maximal rates and using R4.0.1 and RStudio (R Development Core Team, 2009)^1^. Data (*n* = 3) were analyzed using the non-parametric Kruskal–Wallis test. Difference was considered significant at *p* value < 0.05. Principal component analysis (PCA) was calculated using XLSTAT (Pearson correlation matrix).

## Results

### Impact of Pesticides and Metabolites on Potential Denitrification Activity

The impact of pesticides on denitrification was tested in batch conditions in the presence of fungicide (propiconazole) and herbicide (S-metolachlor) or their major metabolites (1,2,4-triazole and ESA-metolachlor, respectively) at two concentrations (2 and 10 μg/L). In presence of acetate and since the last denitrification step was inhibited with the use of acetylene leading to N_2_O accumulation, the denitrification pathway studied is as follows:

$$\mathrm{Step1}:{\text{NO}}_{3}^{-}+1/4{\text{CH}}_{3}{\text{COO}}^{-}+1/4{\text{H}}^{+}\to {\text{NO}}_{2}^{-}+1/2{\text{CO}}_{2\left(\mathrm{aq}\right)}+1/2{\text{H}}_{2}\text{O}$$

$$\mathrm{Step2}:{\text{NO}}_{2}^{-}+1/8{\text{CH}}_{3}{\text{COO}}^{-}+9/8{\text{H}}^{+}\to \mathrm{NO}+1/4{\text{CO}}_{2\left(\mathrm{aq}\right)}+3/4{\text{H}}_{2}\text{O}$$

$$\mathrm{Step3}:\text{NO}+1/8{\text{CH}}_{3}{\text{COO}}^{-}+1/8{\text{H}}^{+}\to 1/2N_{2}\text{O}+1/4{\text{CO}}_{2\left(\mathrm{aq}\right)}+1/4{\text{H}}_{2}\text{O}$$

**FIGURE 1:**
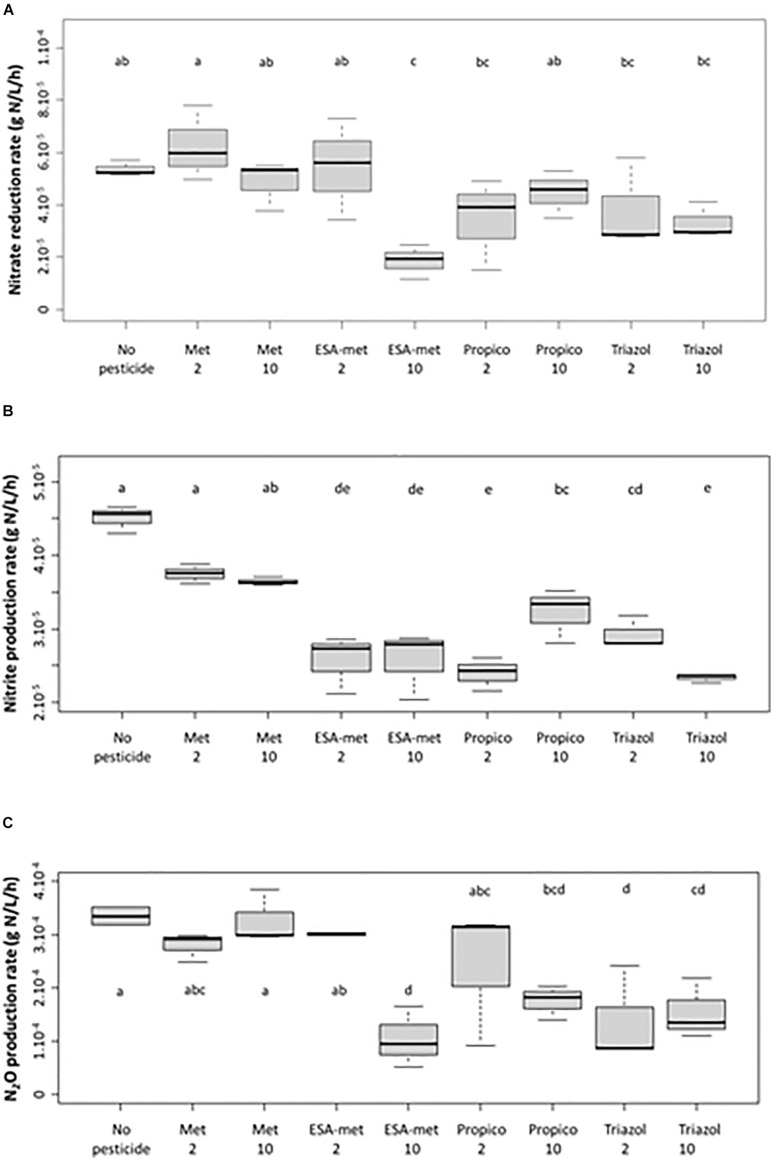
Impact of pesticides and metabolites on the potential denitrification activity (batch experiments) measured as the impact on nitrate (as NO_3_^–^) reduction **(A)**, nitrite (as NO_2_^–^) production **(B)**, and N_2_O production **(C)** rates. Significant differences between conditions were searched applying the Kruskal-Wallis non-parametric test and are mentioned as a, b, c, d, and e letters. No pesticide: control (absence of pesticides or metabolites); Met: metolachlor; ESA-met: ESA-metolachlor Propico: propiconazole; Triazol: 1,2,4 triazole. 2 and 10 are pesticide or metabolite concentrations in μg/L*.* No nitrate reduction nor nitrite and N_2_O production took place in abiotic test batch (data not shown).

**FIGURE 2:**
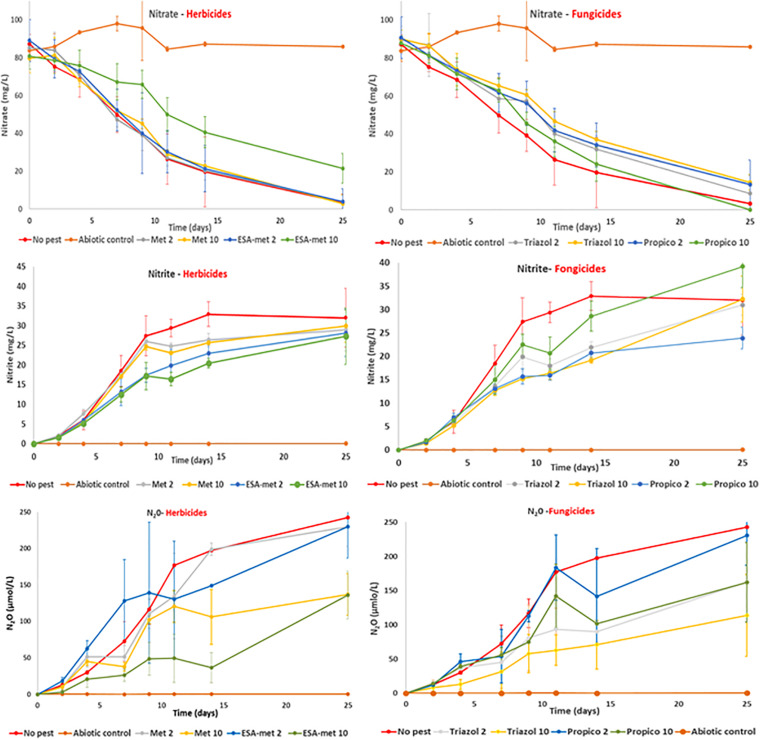
Kinetics of nitrate consumption **(top)**, nitrite production **(middle)**, and N_2_0 production **(bottom)** measured in batch experiments in the absence (no pest) or presence of herbicides [Left: Metolachlor (Met) or its metabolite ESA-metolachlor (ESA-met)] or fungicides [Right: Propiconazole (Propico) or its metabolite 1,2,4-triazole (Triazol)] at 2 or 10 μg/L. Abiotic control were performed in the presence of azide (cf. “Materials and Methods” section).

The following parameters were quantified: nitrate reduction, nitrite production (step 1), and N_2_O production (step 3) rates (Figures 1, 2); these parameters are specific to the denitrification pathway. NO produced during step 2 is a short-lived compound difficult to analyze. Indeed, NO is immediately reduced to nitrous oxide (N_2_O) by cytochrome c (cNOR) or quinone (qNOR) membrane-bound reductases (Santana et al., 2017). For this reason, NO was not quantified and we focused on the other nitrogen-bearing compounds. The nitrogen balance was verified and validated for all the batch experiments with a constant nitrogen concentration all along the experiment (Table 2). No nitrate reduction and no nitrite and N_2_O production were observed in abiotic test flasks.

**TABLE 2:** Nitrogen balance and transformation and acetate concentration during denitrification experiments (batch, *n* = 3) in the presence or absence of pesticides or metabolites (at 2 or 10 μg/L) (here after 7 days of incubation).

Treatment	Nitrogen (after 7 days batch experiments)			Acetate concentration (mg/L) (after 7 days batch experiments)
	As N-NO_3_^–^ (%)	As N-NO_2_^–^ (%)	As N-N_2_O (%)	
Abiotic control	100	0	0	130.1 ± 4.8
No pesticide	54.8 ± 3.4	34.2 ± 0.2	10.9 ± 3.1	48.2 ± 5.5
Metolachlor, 2 μg/L	62.3 ± 4.6	31.1 ± 1.3	6.6 ± 3.4	50.4 ± 4.2
Metolachlor, 10 μg/L	66.2 ± 2.9	29.3 ± 1.9	4.5 ± 2.7	53.3 ± 8.6
ESA-Metolachlor, 2 μg/L	63.2 ± 8.6	21.3 ± 42	15.5 ± 11.6	55.7 ± 8.1
ESA-Metolachlor, 10 μg/L	77.4 ± 5.7	19.6 ± 3.8	3 ± 2	53.2 ± 3.1
Propiconazole, 2 μg/L	72.6 ± 5.3	21 ± 2.3	6.4 ± 5.3	54.4 ± 5.6
Propiconazole, 10 μg/L	69.8 ± 5.3	22.5 ± 4.3	7.5 ± 1.1	66 ± 7.3
1,2,4-triazole, 2 μg/L	72.2 ± 5.5	22.8 ± 1.2	5.1 ± 5.3	55.3 ± 4.9
1,2,4-triazole, 10 | jg/L	76.6 ± 3.3	20 ± 1.6	3.4 ± 3.7	56.4 ± 1.2

*For denitrification activity characterization, rates for nitrate consumption, nitrate production, and N_2_O production were calculated with the data obtained during the 0–7-day period, corresponding to the maximal rates. At T0, nitrogen was present as N-NO_3_ (100%) in all flasks. The nitrogen balance was also verified at all sampling times.*

The nitrate reduction rate measured in the absence of pesticides or metabolites (control) was about 5.6 × 10^–5^ g N/L/h (Figure 1A). With S-metalochlor (at both concentrations) and ESA-metalochlor at 2 μg/L, the kinetic rates were similar. However, ESA-metolachlor at 10 μg/L had the most significant impact on nitrate reduction rate among all the tested conditions, as on average a decrease of 65% was observed. For triazoles, both propiconazole and 1,2,4-triazole decreased the nitrate reduction rate by 29–38% at both concentrations so that high concentrations (10 μg/L) did not result in higher impacts.

In the presence of pesticides or metabolites, a decrease in nitrite production compared with the control (estimated at 4.5 × 10^–5^ g N/L/h) was observed (Figure 1B). With S-metolachlor, at both concentrations, the nitrite production rates decreased by about 10% with respect to the control, but this weak decrease was statistically not significant. With the other compounds, the decrease was significant and could reach almost 50%. As a reminder, the nitrite concentrations measured in flasks correspond to a mean value taking into account nitrite production due to nitrate reduction and nitrite consumption due to nitrite reduction into NO and then N_2_O.

The production rate of N_2_O (Figure 1C) was statistically not affected by the presence of metolachlor and ESA-metolachlor at 2 μg/L since the values were similar to the ones of the control (production close to 3.4 × 10^–4^ g N/L/h). In contrast, N_2_O production rate was negatively affected by the presence of ESA-metolachlor at 10 μg/L (by 70%) and triazole at both concentrations (by 40–70%).

**FIGURE 3:**
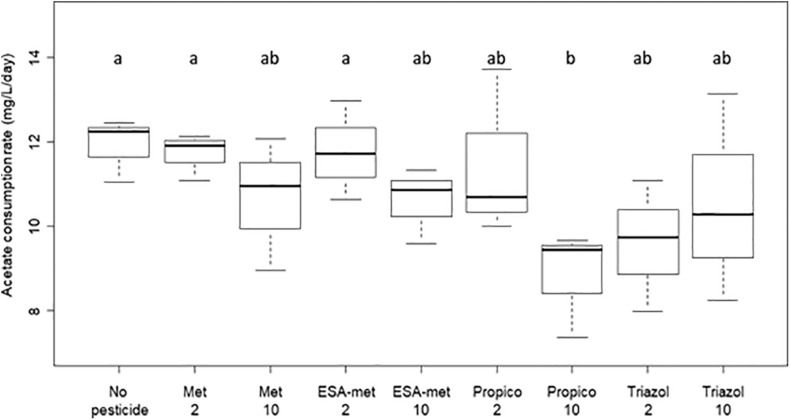
Acetate consumption rate during denitrification in batch experiments with or without pesticides or metabolites. No acetate consumption took place in abiotic test batch (data not shown). No pesticide: control (absence of pesticides or metabolites); Met: metolachlor; ESA-met: ESA-metolachlor; Propico: propiconazole; Triazol: 1,2,4-triazole. 2 and 10 are pesticide or metabolite concentrations in μg/L. Significant differences between conditions were searched applying the Kruskal-Wallis non-parametric test and are mentioned as a and b letters.

All the results suggest that according to the compound and, in some cases, its concentration, denitrification activity was either not affected or negatively impacted (decrease of nitrate reduction rate and nitrite and N_2_O production rates). As mentioned above, the denitrification process in the batch experiments was based on acetate consumption. Acetate was in excess in the solution (Table 2) and its consumption rates in the presence of the tested pesticides and metabolites did not vary significantly from the control (no pesticide), except in the presence of propiconazole at 10 μg/L for which a lower acetate consumption rate was measured (Figure 3). This strongly suggests that there is no direct link between denitrification activity and acetate consumption (in such case, acetate consumption rate would be lowered in conditions such as ESA-metolachlor 10 μg/L).

**FIGURE 4:**
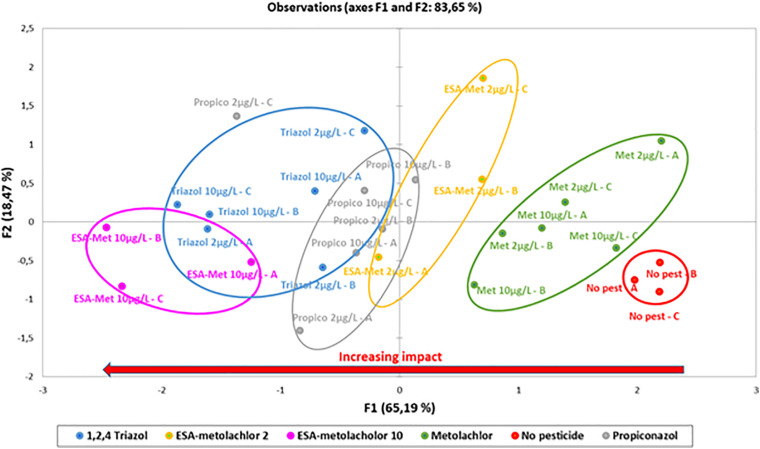
Principal component analysis (PCA) of potential denitrification parameters measured during batch experiments in the presence or absence of pesticides at *2* or 10 μg/L. The percentages of explained variations for the first two axes are indicated within the figure. No pest: control (absence of pesticides or metabolites); Met: metolachlor; ESA-met: ESA-metolachlor; Propico: propiconazole; Triazol: 1,2,4-triazole. *2* and 10 are pesticide or metabolite concentrations in μg/L. A, B, and C are replicates.

The PCA analysis of the parameters used to characterize the potential denitrification activity during batch experiments indicated, according to F1 explaining 65% of the variability (nitrate consumption, nitrite production, and N_2_O production rates all contribute to this axis), the following order for the impact of pesticides and metabolites on denitrification (Figure 4): control (without pesticides or metabolites) < S-metolachor (2 and 10 μg/L) < ESA-metolachlor (2 μg/L) < propiconazole (2 and 10 μg/L) < 1,2,4-triazole (2 and 10 μg/L) < ESA-metolachlor (10 μg/L). ESA-metolachlor at 10 μg/L is thus the condition for which all the denitrification rates (nitrate reduction, nitrite production, and N_2_O production) were the most significantly decreased.

Results from batch experiments also suggested that when denitrification inhibition was observed, at least the first step of denitrification was impacted as nitrate reduction rate significantly decreased (Figure 1A). To determine if pesticides also impacted the other steps of the denitrification pathway, the nitrate reduction rate vs. N_2_O production rate ratio was calculated. Whatever the compound and its concentration, its presence did not modify significantly this ratio (data not shown). This suggested that the lower N_2_O production rates measured in some conditions were directly linked to lower nitrate reduction rates. The impact of the tested pesticides and metabolites thus mainly concerned the first step of denitrification, i.e., nitrate reduction into nitrite.

### Impact of Pesticides and Metabolites on the Bacterial Denitrifying Community

**FIGURE 5:**
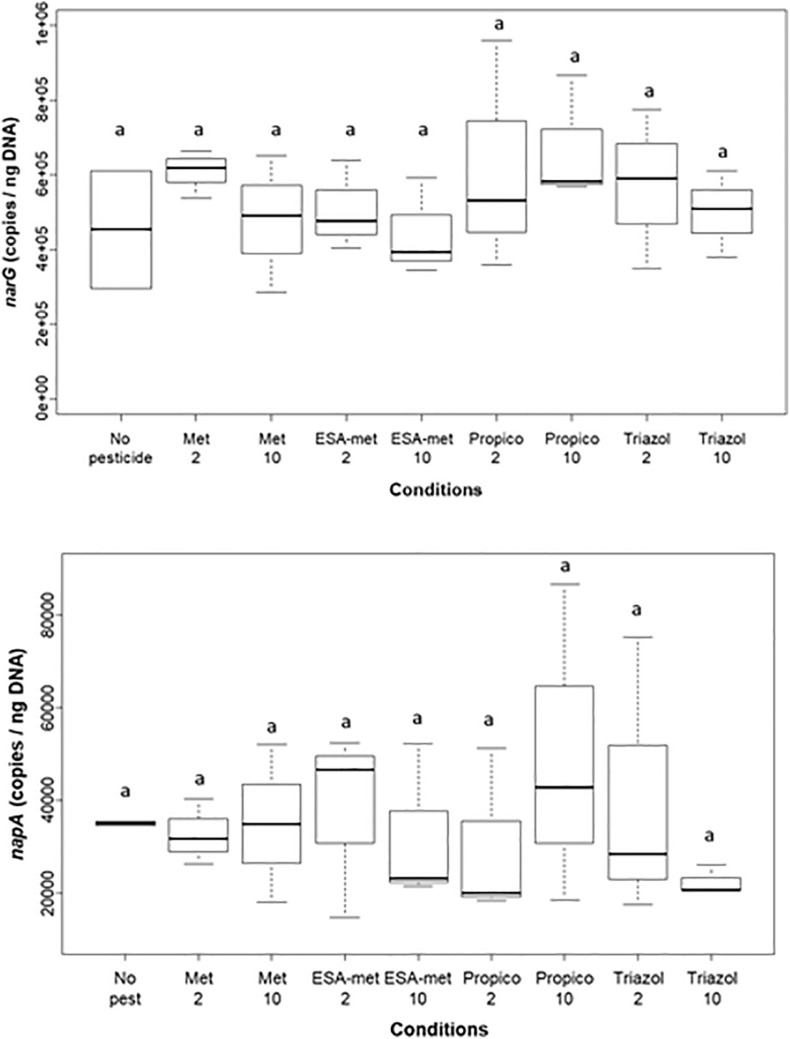
Abundance of *narG* **(top)** and *napA* **(bottom)** genes (involved in nitrate reduction) at the end of batch experiments. Results are given as relative abundance, i.e., data were normalized by taking into account molecular biomass. No pest: no pesticide or metabolite (control); Met: metolachlor; ESA-met: ESA-metolachlor; Propico: propiconazole; Triazol: 1,2,4 triazole. 2 and 10 are pesticide or metabolite concentrations in μg/L. Significant differences between conditions were searched applying the Kruskal–Wallis non-parametric test and are mentioned as a letter.

As the first step (nitrate reduction into nitrite) of the denitrification pathway was shown to be mainly impacted, and in order to determine if the proportion of the denitrifying community changed among the microbial community, the relative abundance of *narG* and *napA* genes, both encoding nitrate reductase, was measured using qPCR approaches (Figure 5). Data were normalized by taking into account molecular biomass (total DNA) (Bru et al., 2007; Wakelin et al., 2011; Hernández-del Amo et al., 2018). The presence of pesticides or metabolites did not significantly modify the relative abundance of both genes even in conditions for which nitrate reduction rate was decreased (in particular ESA-metolachlor at 10 μg/L), suggesting an impact (inhibition) of pesticides and metabolites at the protein level rather than on the nitrate-reducing bacteria abundance within the bacterial community.

### Impact on Microbial Biomass

**FIGURE 6:**
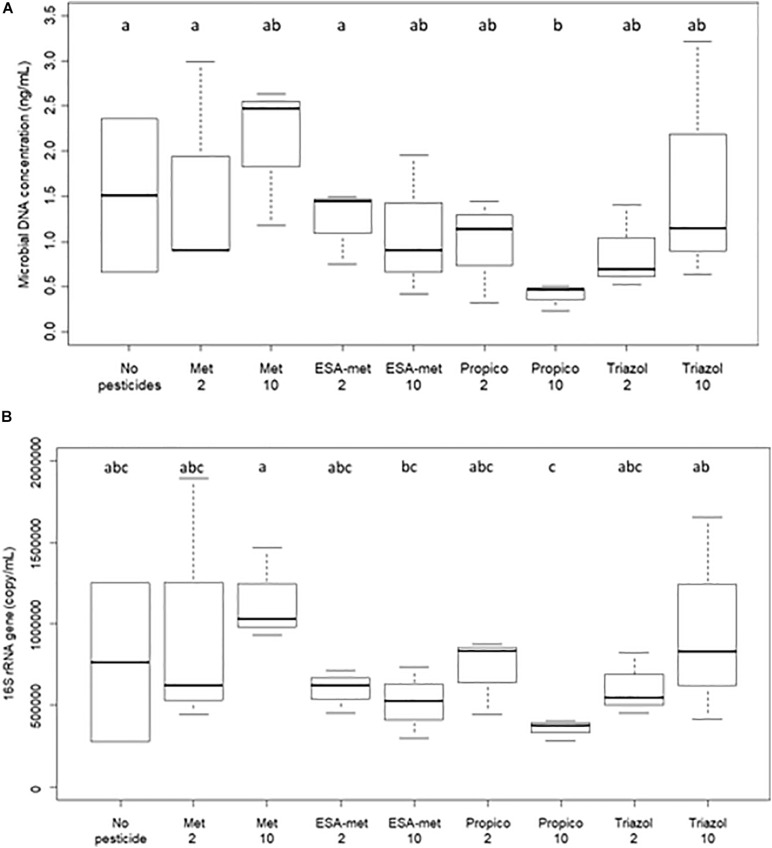
Biomass quantification in flasks at the end of batch experiments. **(A)** Molecular biomass (measured as total DNA concentration) and **(B)** bacterial biomass (measured as bacterial 16S rRNA gene copies). No pesticide: control (absence of pesticides or metabolites); Met: metolachlor; ESA-met: ESA- metolachlor; Propico: propiconazole; Triazol: 1,2,4-triazole. 2 and 10 are pesticide or metabolite concentrations in μg/L. Significant differences between conditions were searched applying the Kruskal–Wallis non- parametric test and are mentioned as a, b, and c letters.

The impact of pesticides and metabolites on groundwater microbial biomass was evaluated at the end of the batch experiments *via* the measure of molecular biomass (total DNA) and bacterial biomass. Both parameters were the same in the presence or absence of pesticides or metabolites whatever the molecule and its concentration with the exception of propiconazole at 10 μg/L. In this condition, a significant decrease in molecular biomass was observed, and the lowest bacterial biomass was measured (Figure 6). This first strongly suggests that there is no direct correlation between denitrification activity and molecular biomass or bacterial biomass (in this case, a decrease of these parameters should have been observed in the ESA-metolachlor 10 μg/L condition particularly). This also suggests that propiconazole at 10 μg/L can inhibit groundwater microbial growth.

### Impact on Bacterial Diversity

**FIGURE 7:**
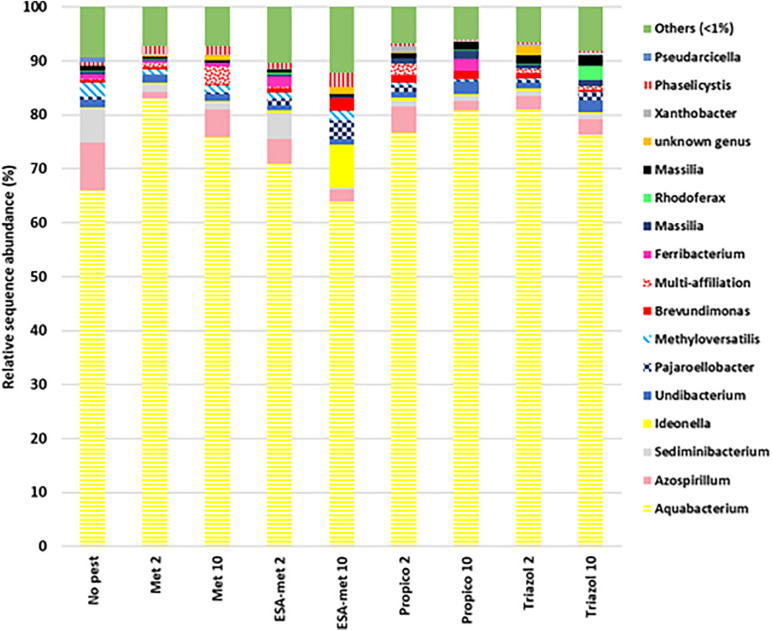
Relative abundance of bacterial genera at the end of batch experiments, representing at least 1% of obtained sequences in at least one sample. Data represent average values of experimental replicates. No pest: control (absence of pesticides or metabolites); Met: metolachlor; ESA-met: ESA-metolachlor; Propico: propiconazole; Triazol: 1,2,4-triazole. 2 and 10 are pesticide or metabolite concentrations in μg/L.

**FIGURE 8:**
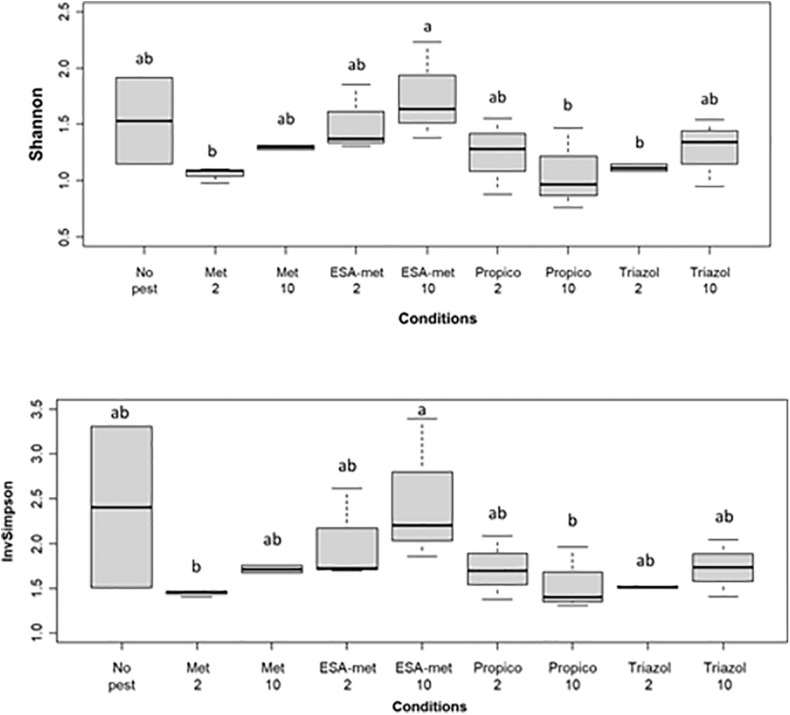
Biodiversity index of the bacterial communities at the end of batch experiments (20 days): **(Top)** Shannon index and **(Bottom)** InvSimpson index. No pest: control (absence of pesticides or metabolites); Met: metolachlor; ESA-met: ESA-metolachlor; Propico: propiconazole; Triazol: 1,2,4-triazole. 2 and 10 are pesticide or metabolite concentrations in μg/L. Significant differences between conditions were searched applying the Kruskal-Wall is non-parametric test and are mentioned as a, b, and c letters.

The bacterial community diversity was determined by Illumina 16S rRNA sequencing on batch experiment samples after pesticide or metabolite exposure during 20 days. Results first showed that among all the samples, the two main phyla were Proteobacteria (as the most dominant phylum) and to a lesser extent Bacteroidetes. As expected, when looking at the genus level, the conditions applied in batch experiments in favor of denitrification led to the selection of bacteria involved in the N cycle. Indeed, the *Aquabacterium* genus, known to reduce nitrate into nitrite (Kalmbach et al., 1999), was the most dominant genus at the end of all batch experiments (Figure 7). In addition, other genera such as *Azospirillum* (Kloos et al., 2001) and *Rhodoferax* (Hougardy and Klemme, 1995), also known to reduce nitrate into nitrite, were detected. No genus was specific to a given batch condition except *Ideonella* that was found only in the condition with ESA-metolachlor at 10 μg/L and *Rhodoferax* found in the condition with 1,2,4-triazole at 10 μg/L. There was no significant difference between values of Shannon and InvSimpson (1/*D*) biodiversity indices among most samples (Figure 8), suggesting that pesticides and metabolites at low (environmental) concentrations did not significantly impact groundwater bacterial community diversity. However, results suggested that ESA-metolachlor at 10 μg/L tended to increase Shannon and InvSimpson indices and that propiconazole at 10 μg/L tended to decrease both biodiversity indices. When comparing diversity depending on the pesticide or metabolite type and concentration, bacterial communities were comparable in terms of genus presence and relative abundance tendencies, except for the conditions with ESA-metolachlor at 10 μg/L and 1,2,4-triazole at 10 μg/L, in which *Ideonella* and *Rhodoferax* were specifically found, respectively, as mentioned above.

## Discussion

In this paper, the potential side effects of some pesticides and their metabolites frequently detected in groundwater on groundwater microbial ecosystems were investigated. In particular, a focus was done on the potential impact of these compounds on the denitrification activity supported by the groundwater bacterial community. The strategy used in the study was to perform all the tests (batch experiments at the lab scale) with the same groundwater sample, which allowed comparing the results. Indeed, such a strategy enables to circumvent the potential effects due to hydrogeochemical and seasonal variations in groundwater and to strong spatial and temporal variations in groundwater bacterial communities (de Lipthay et al., 2004; Imfeld et al., 2018). In our study, nitrate reduction rate measured in the absence of pesticides/metabolites (control) was about 5.6 × 10^–5^ g N/L/h. Even if it is difficult to compare acquired data with already published denitrification rates because of the numerous factors affecting this process (pH, temperature, nitrate and electron donor concentrations, etc.), denitrification rates measured in laboratory experiments generally vary between 7.0 × 10^–6^ and 1.0 × 10^–4^ g N/L/h (Trudell et al., 1986; Starr and Gillham, 1993; Schipper and Vojvodic Vukovic, 1998, 2000; Devlin et al., 2000; André et al., 2011). The values determined in this study for the control (without pesticide) and for the conditions with pesticide/metabolite are thus in the same order of magnitude than other studies.

### Side Effects of Pesticides and Their Metabolites on the Metabolism of Groundwater Microbial Community

Among the compounds tested, S-metolachlor at environmental concentrations was shown to have no or minor impact on denitrification as well as on general microbial parameters (biomass, acetate consumption) of the tested groundwater microbial community. This is in agreement with previous studies such as that of de Lipthay et al. (2004) who suggested no major effect of S-metolachlor on *in situ* groundwater bacterial community (using Ecolog and DGGE fingerprinting approaches). Mauffret et al. (2017) showed that the chloroacetanilide family had no impact on the bacterial community structure but increased the abundance of nitrate reductase *napA* gene. This last point is not in agreement with our results that showed no impact of the tested pesticides on the relative abundance of the nitrate reductase *narG* and *napA* genes involved in the first step of denitrification. One explanation is probably that the data of Mauffret et al. (2017) were obtained from *in situ* groundwater, whereas our results concern batch experiments, and it has been demonstrated that for *in situ* studies particularly, there are difficulties to tease apart the effect of pesticides from the effects of variations of hydrogeological conditions (Imfeld et al., 2018). Moreover, as mentioned above, microbial community diversity as well as the history of pesticide contamination (i.e., communities previously in contact or not with pesticides) can influence the impact of pesticides. Finally, the work of Imfeld et al. (2018) also suggested a little effect of metolachlor on *in situ* bacterial groundwater community (Microtox test and T-RFLP fingerprints). In this same work, the transformation products of S-metolachlor (as ESA and OXA-metolachor) were shown to have no effect on microbial metabolism. We obtained a similar result in denitrifying condition when considering general growth parameters. Indeed, ESA-metolachlor had no impact on biomass and acetate consumption. However, in such anoxic conditions, ESA-metolachlor (at 10 μg/L) had the highest negative impact on denitrification. Taking into account our study and that of Imfeld et al. (2018), it can be suggested that pesticides or metabolites (like ESA-metolachlor) could mainly have side effects on specific communities and/or targeted metabolisms (like denitrification), and not on the whole microbial community.

Concerning the potential side effects of the members of the conazole family, our results suggested that propiconazole and 1,2,4-triazole had a higher adverse effect on the microbial denitrifying community of the studied groundwater than chloroacetanilide [as S-metolachlor and ESA-metolachlor (at 2 μg/L)]. Moreover, propiconazole (at 10 μg/L) was shown to inhibit microbial growth as less microbial biomass was produced in its presence. This is in agreement with Milenkovski et al. (2010) who found (via leucine incorporation experiments) that propiconazole and other fungicides belonging to different chemical classes had an inhibitory effect on microbial metabolism. In their study, these authors did not notice any impact of propiconazole on denitrification but measured an inhibition of the denitrification pathway with other fungicides. Saez et al. (2003), by comparing the impact of various insecticides, herbicides, and fungicides (at 10 μg/L) on the pure denitrifying strain *Paracoccus denitrificans*, observed the more severe effects on bacterial growth and activity with fungicides. Staley et al. (2015) also concluded, by comparing a large number of studies, that fungicides seemed to be most deadly to microorganisms than herbicides.

At last, this study showed that among the tested compound, metabolites (here, ESA-metolachlor and 1,2,4-triazole) have a more significant impact on denitrification than mother molecules (Figure 4). Bollag and Kurek (1980) already demonstrated that chlordimeform (organochloride insecticide) metabolites (at 25 mg/L and more) negatively impacted soil microbial denitrification (accumulation of nitrite and nitrous oxide), whereas chlordimeform itself had no impact at the same concentrations (and even at 100 mg/L). As mentioned in the *Introduction*, metabolites exhibit high occurrence in groundwater and sometimes with concentrations of the order of micrograms per liter. This was demonstrated by several studies such as Fisher et al. (2021) for ESA-metolachlor and 1,2,4-triazole as well as for other metabolites such as 4-hydroxychlorothalonil (ranging from 0.13 to 368 μg/L). Another recent study in which 116 metabolites on 1,204 sites were analyzed also showed that six metabolites exhibit concentrations higher than 0.1 μg/L [notably 4-hydroxychlorothalonil in 1.6% of the samples with a maximum concentration of 17.540 μg/L, and 22 metabolites with a detection frequency higher than 1% (Bexfield et al., 2021)]. Our results together with other studies thus suggest that metabolites can greatly impact the microbial ecosystem in groundwater.

### Side Effects of the Tested Pesticides and Metabolites on the Denitrification Pathway

Our study showed that the first step of denitrification (reduction of nitrate into nitrite) supported by nitrate reductase enzymes was the main step impacted/inhibited by the studied pesticides or metabolites. Nitrate reductase is encoded by *narG* or *napA* genes, depending on the bacterial strain. In our study, the abundance of *narG* and *napA* genes was not impacted by the presence of the tested pesticides and metabolites. This suggests that the impact of the tested compounds probably occurs rather on gene expression and/or enzymatic activity itself. Pankaj and Gore (2015) already showed that the activity of enzyme nitrate reductase of plants was inhibited by some phytopharmaceutical products (confidor, omite, and karathane) and their active substances (imidacloprid, propargite, and meptyldinocap). The work of Saez et al. (2003) also suggested that some pesticides (methylparathion, atrazine, and simazine) affected the expression of the nitrate reductase activity of the bacterial strain *P. denitrificans* at a concentration of 10 mg/L. The activity of other enzymes of the denitrification pathway was shown, in some cases, to be also affected by pesticides. As an example, the presence of aldrin, lindane, dimethoate, and methidathion led to the inhibition of nitrite reductase activity in the study conducted by Saez et al. (2003). Su et al. (2019) also observed (in riparian sediments) an inhibition of the activity of nitrate reductase, nitrite reductase, and N_2_O reductase (but not NO reductase) enzymes involved in the denitrification pathway by the pesticide chlorothalonil (at 2 mg/kg and more). These authors also demonstrated an impact of this pesticide on *narG* gene relative abundance, but not on the relative abundance of *nirK*, *norB*, and nosZ genes involved in the next steps of denitrification. This indicates that there was no direct matching between responses to the presence of pesticides at the protein and genetic levels. In our study, only nitrate reductase activity was impacted and this impact was detected for concentrations as low as 2 or 10 μg/L according to the pesticides or metabolites (Figure 1). However, it has to be mentioned that as we used acetylene in our experiment, the impact of pesticides or metabolites on the last step of denitrification (reduction of N_2_O into N_2_ by the enzyme nitrous oxide reductase encoded by *nosZ* gene) cannot be evaluated here.

### Diversity of the Groundwater Bacterial Community in the Presence of Pesticides and Metabolites

In our study, the presence and relative abundance of bacterial genera were similar for most of the conditions, suggesting that the composition of the bacterial community was generally weakly affected by the presence of pesticides or metabolites at environmental concentrations. Only two genera were found specific (*Ideonella* for ESA-metolachlor at 10 μg/L and *Rhodoferax* for 1,2,4-triazole at 10 μg/L). This is in agreement with previous studies that reported minor effects of pesticides on the overall composition of the groundwater microbial community. Indeed, Imfeld et al. (2018) found that OTUs specific to pesticide presence and correlating with metolachlor (even at 5 mg/L) addition in batch experiments ranged only between 0.4 and 3.6% of the total. In the same way, de Lipthay et al. (2004) found no DGGE DNA bands that were unique for the herbicide (a mixture of six herbicides at 40 μg/L each)-exposed subsurface sediments (shallow aquifer). Shannon and InvSimpson diversity indices obtained at the end of our batch experiments suggested a potential increase or decrease of bacterial diversity in the presence of ESA-metolachlor or propiconazole at 10 μg/L, respectively. On the opposite, ESA-metolachlor and propiconazole at 2 μg/L, as well as the other tested pesticides and metabolites, did not impact diversity indices suggesting that the diversity may be impacted differently according to the molecule and its concentration. Results obtained with ESA-metolachlor at 10 μg/L are in agreement with several other studies on groundwater impacted by pesticides that, as mentioned above, displayed higher diversity indices [based on carbon source utilization (EcoPlate), colony morphology, DGGE analyses, or Illumina sequencing] in the presence of some pesticides (de Lipthay et al., 2004; Janniche et al., 2012; Imfeld et al., 2018). The higher diversity observed in some pesticide-polluted aquifers compared with unpolluted aquifers can be explained by various hypotheses. First, as some microorganisms can metabolize pesticides and use them as a source of C (Munoz et al., 2011; Satapute and Kaliwal, 2016) and even N (Sanchez-Sanchez et al., 2013; Wang et al., 2016) or P (Shushkova et al., 2012) according to pesticide composition, the presence of pesticides can result in an increase in microbial abundance and diversity. This is particularly true in the case of groundwater that is naturally weakly charged in organic matter [as the groundwater we used in this study, whose DOC content was under the limit of detection (Table 1)] and where pesticides can represent a new source of nutrients. Secondly, as pesticides have a lethal effect on some microorganisms but not on all microorganisms, their presence can lead to the cell lysis of non-resistant microorganisms and thus increase nutrient sources for resistant microorganisms and thus impact biodiversity. Thirdly, the presence of a toxic compound, by killing sensitive strains and/or inhibiting (even partially) their activity, limits the number of competitors for available nutrients. Resistant microorganisms can thus thrive in an easier manner, and strains initially in minority, if resistant, can become dominant strains. This could be the case in batch experiments with ESA-metolachlor (10 μg/L) in our study. Indeed, this metabolite partially inhibits and slows down the activity of denitrifying bacteria, and its presence allows the development of microorganisms such as *Ideonella*, *Brevundimonas*, and *Pajaroellobacte*r that are in higher abundance or even specific to this condition and that are not known to be linked to denitrification, which leads to a higher diversity. This is in agreement with Staley et al. (2015) who concluded that in a microbial community, even if the direct effects of a pesticide family on some microorganisms are negative, some indirect positive effects can occur for other microorganisms. The direct negative effects can thus tend to be offsetted when considering the impact at the community level. These authors also insist on the fact that it is difficult to conclude on the direct impacts of pesticides on bacterial communities as the impact can be contrasting according to bacterial species, pesticide concentration, time of exposure, etc., probably due to the broad diversity of strategies used by bacteria. Finally, as underlined by Jacobsen and Hjelmsø (2014), conclusions on the direct and/or indirect effects induced by the presence of pesticides on microbial diversity are not easy to determine. Indeed, in most cases, the links between phylogeny and functions are not directly established for bacteria, and because in all ecosystems, many groups (such as – according to the ecosystem – bacteria, archaea, fungi, plants, and nematodes) interact within or between each other.

## Conclusion

The current study demonstrates that some pesticides and metabolites can have a negative impact on the activity of the denitrifying community in groundwater (decrease of the activity) at environmental concentrations. The impact of metabolites can be higher than that of pesticides (parent molecules) and mainly concerns the first step of the denitrification pathway, very probably because of an inhibitory impact on the nitrate reductase enzyme itself. Finally, the presence of some pesticides and metabolites in groundwater can also impact the global growth of the groundwater microbial community as well as its biodiversity. The study thus emphasizes that pesticides and metabolites affect groundwater communities even at environmental low concentrations (2 μg/L and potentially less), strongly insisting on the need to carry out studies at low pesticide/metabolite concentrations to get a realistic picture on how these molecules can affect subsurface microbial communities and activities.

This study, with a few others, is of particular interest as the side effects of pesticides and metabolites on soil microorganisms already have to be taken into account during European pesticide registration (regulation EC no. 1107/2009). Indeed, approved pesticides shall have no unacceptable effects on the environment, e.g., impact on soil biodiversity and the ecosystem. To date, scientific methods accepted by the authority are available for studying the impact on N cycle in soils (test guideline OCDE 216 “Soil Microorganisms: Nitrogen Transformation Test”), thus allowing a systematic evaluation for each new approved (or re-approved) active substance. Our results illustrate the importance to also consider more systematically the impacts on subterranean ecosystems in the frame of the European pesticide registration procedure. Finally, the impacts of pesticides and/or metabolites as cocktail will have also to be taken into account in future evaluations of pesticides for their registration.

## Data Availability Statement

The datasets presented in this study can be found in online repositories. The names of the repository/repositories and accession number(s) can be found below: https://www.ebi.ac.uk/ena, PRJEB42682.

## Author Contributions

CM, NB, LA, and CJ conceived and designed the research. CM and CJ conducted the experiments, with the technical support of MC, and were actively involved in microbial analyses. NB was involved in sampling site chemical characterization and groundwater sampling as well as chemical analysis. LA was involved in chemical and nitrogen balance analysis. CM wrote and revised the manuscript. NB, LA, and CJ were actively involved in its editing and revision. All authors read, corrected, and approved the manuscript.

## Conflict of Interest

The authors declare that the research was conducted in the absence of any commercial or financial relationships that could be construed as a potential conflict of interest.

## Footnotes

1 http://www.R-project.org
